# Carbohydrate restricted diet in conjunction with metformin and liraglutide is an effective treatment in patients with deteriorated type 2 diabetes mellitus: Proof-of-concept study

**DOI:** 10.1186/1743-7075-8-92

**Published:** 2011-12-23

**Authors:** Jürgen E Müller, Dagmar Sträter-Müller, Hans-Joachim Marks, Michael Gläsner, Philipp Kneppe, Beate Clemens-Harmening, Harald Menker

**Affiliations:** 1 Internistische Praxis, Bödefelderstr. 8, Schmallenberg, Germany; 2 Internistische Praxis, Am Bahnhof 4-12, Siegen, Germany; 3 Internistische Praxis, Bahnhofstr. 28, Netphen, Germany; 4 Internistische Praxis, Am Baumhof 6, Wenden, Germany; 5 Internistische Praxis, Neumarkt 14, Netphen, Germany

## Abstract

**Background:**

Type 2 diabetes mellitus is a chronic progressive disease. During the course of the disease intensive treatment is often necessary resulting in multiple interventions including administration of insulin. Although dietary intervention is highly recommended, the clinical results of the widely prescribed diets with low fat content and high carbohydrates are disappointing. In this proof-of-concept study, we tested the effect of dietary carbohydrate-restriction in conjunction with metformin and liraglutide on metabolic control in patients with type 2 diabetes.

**Methods:**

Forty patients with type 2 diabetes already being treated with two oral anti-diabetic drugs or insulin treatment and who showed deterioration of their glucose metabolism (i.e. HbA1c *>*7.5), were treated. A carbohydrate-restricted diet and a combination of metformin and liraglutide were instituted, after stopping either insulin or oral anti-diabetic drugs (excluding metformin). After enrollment, the study patients were scheduled for follow-up visits at one, two, three and six months. Primary outcome was glycemic control, measured by HbA1c at six months. Secondary outcomes were body weight, lipid-profile and treatment satisfaction.

**Results:**

Thirty-five (88%) participants completed the study. Nearly all participating patients experienced a drop in HbA1c and body weight during the first three months, an effect which was maintained until the end of the study at six months. Seventy-one percent of the patients reached HbA1c values below 7.0%. The range of body weight at enrollment was extreme, reaching 165 kg as the highest initial value. The average weight loss after 6 months was 10%. Most patients were satisfied with this treatment. During the intervention no significant change of lipids was observed. Most patients who were on insulin could maintain the treatment without insulin with far better metabolic control.

**Conclusions:**

Carbohydrate restriction in conjunction with metformin and liraglutide is an effective treatment option for patients with advanced diabetes who are candidates for instituting insulin or who are in need of intensified insulin treatment. This proof-of-principle study showed a significant treatment effect on metabolic control.

## Background

Diabetes and obesity are closely linked diseases with rising prevalence and incidence in developed and developing countries [1]. Westernized eating habits and lifestyle are presumed to be the major reasons for this epidemic. Official guidelines recommend diets with low fat contents and high amounts of carbohydrates although it has never been proven that these are effective in reducing cardiovascular disease morbidity and mortality, the major health problems connected with diabetes and obesity [2]. Recently the Women's Health Initiative study showed no effect of a diet restricted in fat content and enriched in carbohydrates on cardiovascular morbidity and mortality [3]. Reducing body weight is considered an important therapeutic intervention to treat patients with type 2 diabetes and is a major challenge in ambulatory settings. Most intervention trials have failed to demonstrate a long lasting effect. Putting patients on insulin will often cause an increase in body weight resulting in the need for further insulin to be injected. Although many patients are on high insulin doses, metabolic results are often poor with high HbA1c values. The standard low fat-high carbohydrate intervention has been challenged as proper interventions in obese patients and avoiding "fattening" carbohydrates is recommended as a strong alternative therapeutic option.

Historically, there has been a scientific tradition favoring dietary carbohydrate-restriction in obese patients in Europe before the Second World War [4] and in the Fifties in the USA [5]. The recently published A to Z trial showed that the most beneficial effect in weight reduction was in those patients treated with a carbohydrate-restricted diet in comparison with three other dietary interventions, lower in fat and higher in carbohydrates [6]. There may be individual differences, some patients doing better on a carbohydrate-restricted diet to reduce weight while others do better on a fat-restricted diet. A possible explanation might be a different insulin response after a glucose challenge, i.e. a high response reflecting insulin resistance [7], in those patients that did better on a carbohydrate-restricted diet.

In addition, diets that are rich in carbohydrates will result in an unfavorable cardiovascular risk profile resulting in raised triglycerides and lowered HDL and increased small dense LDL [8]. Glycemic index of carbohydrates is a strong determinant of HDL-cholesterol concentration in plasma [9]. In context of carbohydrate-restriction, dietary saturated fat has also been shown to exhibit a beneficial effect on plasma lipids [10]. These latter conditions are neglected in official recommendations. A recent review of the scientific evidence of dietary carbohydrate-restriction in type 2 diabetes challenged the official recommendation of low fat diets [11]. The beneficial effect of a low-carbohydrate, ketogenic diet versus a low-glycemic index caloric restricted diet improved metabolic control in patients with type 2 diabetes and also resulted in greater weight loss and reduction or complete cessation of anti-diabetic medications [12]. A two year randomized trial comparing low carbohydrate diet versus low fat diet in obese patients did also show a more favorable lipid profile in those patients randomized to the low carbohydrate diet treatment [13].

The recently introduced GLP-1 analogues have expanded our possible therapeutic options for type 2 diabetes. It has been shown that these analogues, e.g. exenatide and liraglutide, are effective in reducing metabolic outcomes used alone or in combination with oral drugs. It was also shown that liraglutide was as effective as glargine insulin in reducing HbA1c [14]. Bypassing glucose- dependent endogenous insulin stimulation, thus avoiding hypoglycemia, is an important effect of this new class of drugs. In addition to stimulation of insulin release, one major effect of all GLP-1 analogues is the induction of satiety by delaying gastric output. This will facilitate weight reduction, which has been shown for obese non diabetic patients in an intervention trial [15].

## Methods

### Study-design

This study was a 24 weeks multi-center intervention trial, designed as proof-of-concept study without control group. Five medical offices specializing in diabetes care participated in this study after training in the concept of carbohydrate restricted diets and introduction to the educational material to be given to patients. The trial was conducted between 1 September 2010 and 31 July 2011. This trial was conducted in accordance with International Conference on Harmonization guidelines and the Declaration of Helsinki and Good Clinical Practice guidelines [16].

### Participants

Forty ambulatory patients with type 2 diabetes showing unsatisfactory metabolic control of their glucose metabolism, who were either already on two antidiabetic drugs and were scheduled to switch to insulin treatment or who were already on insulin treatment, but were scheduled for intensification of therapy, were included in this study. The inclusion of patients was up to the choice of the participating medical offices. Inclusion criteria were confirmed type 2 diabetes mellitus, HbA1c *>*7.5% and BMI above 30 *kg*/*m*^2^. Exclusion citeria were significant co-morbid diseases such as kidney disease with glomerular filtration rate below 60 ml or liver disease (AST or ALT twice above the upper normal range). There were no incentives paid to the patients.

### Intervention

The patients were introduced to this treatment option and gave informed written consent. After being selected, basic anthropometric measures, i.e. body weight, height and age, were recorded. Basic anamnestic questions regarding duration of diabetes and cardiovascular diseases were asked. Routine laboratory values were taken at study entry. Participants were taught about the principles of carbohydrate restriction diets, received an educational booklet and were trained to inject liraglutide. During the first 2 months patients were counseled to adhere to this diet, which contained less than 20 grams of carbohydrates per day but was unrestricted with respect to calories. After 8 weeks patients were allowed to add small amounts of carbohydrates for breakfast. The starting dose of liraglutide was 0.6 mg/day with adjustment to 1.2 mg after one week until the first control visit four weeks later. Further adjustments were the decision of the participating physician.

Patients were invited to return to the study center for follow-up visits after 1, 2, 3 and 6 months. At all follow up visits laboratory control values including blood glucose, HbA1c, lipids and creatine were measured. Body weight was estimated to the nearest kg. At each visit a physician reviewed the blood glucose values and discussed adjustment of medications.

### Participants Satisfaction with the treatment

During the follow up visits and at the end of the 6-month intervention period, participants could comment on treatment satisfaction in a questionnaire using a 7-point Likert scale. Questions addressed the overall satisfaction with the treatment, weight loss, blood glucose values, the avoidance of carbohydrates and the issue of staying on a low-carb diet. The issue of importance to avoid insulin treatment and avoiding carbohydrates for breakfast, lunch and dinner was evaluated on a 5-point scale separately. The scales were coded 1 = strongly disagree going up to 7 = completely agree for the 7 point scale and 1 = not important at all to 5 = very important for the 5 point scale.

### Statistical analysis

All data were collected anonymously in a data base in the coordinating study center using Epi Info version 3.5.3 for data management http://www.cdc.gov/epiinfo. All statistical analyses were done using SAS 9.0 (SAS Institute Inc, Cary, North Carolina) or R software version 2.92 (R Foundation for Statistical computing, http://www.r-project.org. Analysis of continuous outcome data was done taking repeated measurements into account by using multilevel modeling. Because of unequal intervals between follow up visits, time was additionally used as categorical variable. In addition, analysis of continuous outcome data were done by imputing missing values under the assumption of last observations carried forward (LOCF). P-values *<*0.05 were considered to be significant. Additionally primary outcome was analyzed using a paired t-test or Wilcoxon test as appropriate comparing baseline value and 24-weeks value in complete cases.

### Role of funding source

The study was not funded by external sources. No potential conflicts of interest relevant to this article are reported.

## Results

### Participants

40 patients were initially found eligible for study and agreed to participate. There were 5 drop-outs: One patient did not come to the first follow up visit for unknown reasons and could not be contacted, two people were not able to stay on the diet and two people had to switch back to insulin because of deterioration of blood glucose, leaving 35 patients to be included in the study. 24 patients of the initial cohort were on oral anti-diabetic medication and 16 patients were on insulin. The baseline characteristics of the entire group are shown in Table 1. In Table 1, patients are divided into those on oral drugs or on insulin. There was no difference in age between the two groups. Twenty-three patients were male and 17, female. As expected, duration of diabetes was different between these two groups. The mean duration was 10.3 years in the insulin-treated group and 5.7 years in the group treated with oral anti-diabetic medication.

**Table 1:** Baseline characteristics of participants

	Whole	Group n = 40	Insulin	Group n = 16	OAD †	Group n = 24
	Mean	SD	Mean	SD	Mean	SD
HbA1c	9.0	1.2	8.5	0.8	9.5	1.3
Body weight, kg	116.1	20.2	115.6	22.0	116.4	19.5
Age years	57.1	9.6	56.8	9.7	57.4	9.8
Diabetes Duration	7.5	6.1	10.3	7.8	5.7	3.9
Cholesterol	209.1	44.8	210.0	46.0	208.5	45.1
Triglycerides	254.7	123.5	263.4	118.7	248.9	129.2
HDL-cholesterol	49.5	18.5	47.1	11.6	51.1	22.1
LDL-cholesterol	119.0	35.9	119.6	38.9	118.4	40.1

†OAD: Oral Antidiabetic Drug

### Outcomes

#### Hemoglobin A1c

The average baseline HbA1c was 9.0% for the entire group. The insulin treated group showed a HbAc1 of 8.5%, lower than the HbA1c value for the oral medication-treated patients whose mean HbA1c was 9.5% (Table 1).

**Figure 1:**
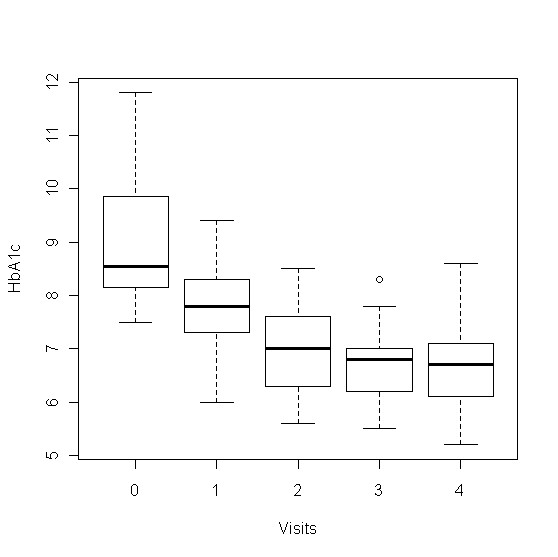
**Time course of HbA1c**. This figure displays the drop of HbA1c during the intervention at all visits until the end of the study at 6 months.

**Figure 2:**
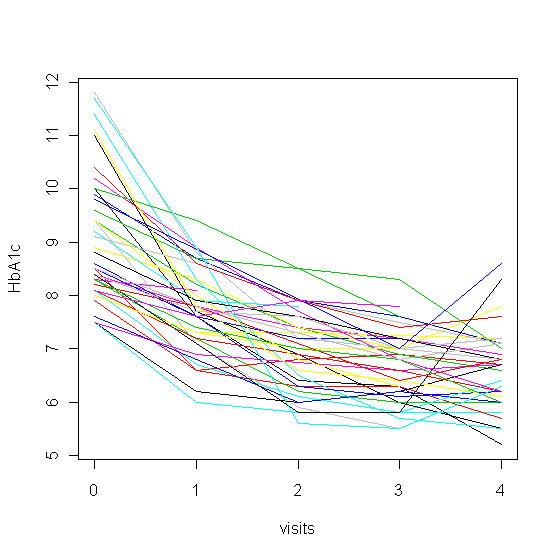
**Trajectories of HbA1c in individual patients**. The pattern in this figure is concerned with individual changes of HbA1c during the intervention. Each line displays the individual change of HbA1c.

**Figure 3:**
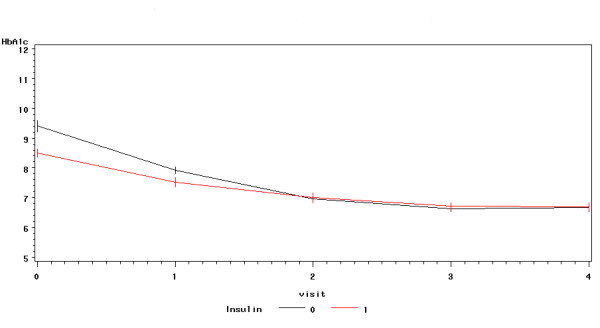
**Time course of HbA1c split by pretreatment**. This figure displays the drop of HbA1c during the intervention at all visits until the end of the study at 6 months split by pretreatment (Insulin versus Oral Antidiabetic Drugs).

As shown in Figure 1, there was a drop of mean HbA1c from baseline value of 9.0% to 6.7% at 24 weeks for the group as a whole (9.0% ± 1.2 to 6.7 ± 0.8%, p < 0.0001, within group change). The individual time course of HbA1c values during the study showed a consistent fall in all but two study participants (Figure 2). The initial HbA1c for insulin treated patients dropped from 8.5% ± 0.8% to 6.7 ± 0.7%, p < 0.0001 at 24 weeks and from 9.5% ± 1.3% to 6.7 ± 0.9%, p < 0.0001 for patients on oral drugs (Table 2). The average decline in HbA1c was nearly superimposable at 2, 3 and 6 months (Figure 3). The absolute drop of HbA1c was greater for patients who had been on oral drugs in comparison to the group who had been on insulin, 2.8% versus 1.8%. In a multilevel model describing HbA1c as dependent variable, the predictor time was highly significant at all time points (p < 0.0001), using time as categorical variable (drop of HbA1c at: Visit 4 = -2.3308, Visit 3 = -2.3583, Visit 2 = -2.0816, Visit 1 = -1.2916) or as continuous variable (drop of HbA1c per time unit = -0.5964). The model with best fit to the data was derived using a random intercept and random slope model. The addition of gender to the model showed no significant effect, but adding insulin resulted in a marginally significant interaction term time*insulin -0.2255 (p = 0.047).

**Table 2:** Effect of treatment program on glycemic control, body weight and plasma lipids

	Week 0 Mean ± SD	Week 4 Mean ± SD	Week 8 Mean ± SD	Week 12 Mean ± SD	Week 24 Mean ± SD
HbA1c	9.0 ± 1.2	7.8 ± 0.8	6.9 ± 0.8	6.6 ± 0.7	6.7 ± 0.8 †
Weight	116.1 ± 20.2	109.9 ± 18.2	108.9 ± 19.0	104.6 ± 17.2	101.3 ± 17.8 †
Cholesterol	209.1 ± 44.8	197.6 ± 41.3	195.7 ± 39.0	197.6 ± 35.0	193.0 ± 32.7 ‡
Triglycerides	254.7 ± 123.5	206.3 ± 77.5	194.5 ± 60.5	198.3 ± 63.5	198.8 ± 92.6 ‡
HDL-cholesterol	49.5 ± 18.5	46.9 ± 17.1	49.7 ± 17.3	51.9 ± 16.9	54.0 ± 17.6 ‡
LDL-cholesterol	119.0 ± 38.9	108.9 ± 32.2	110.8 ± 31.9	107.6 ± 31.6	101.1 ± 29.3 ‡

†p < 0.0001 versus baseline

‡p *>*0.05 versus baseline

#### Body weight

The mean initial body weight was 116.1 kg with a wide range going up to 165 kg. During the time course of the intervention the mean body weight at 24 weeks dropped significantly to 101.3 ± 17.8 kg, p < 0.0001. The drop in body weight in patients who had been on insulin before was greater in comparison with the patient who had been on oral drugs, 115.6 ± 22.0 kg to 94.7.0 ± 17.5 kg versus 116.4 ± 19.5 to 105.3 ± 17.1 kg.

#### Lipid parameters

**Figure 4:**
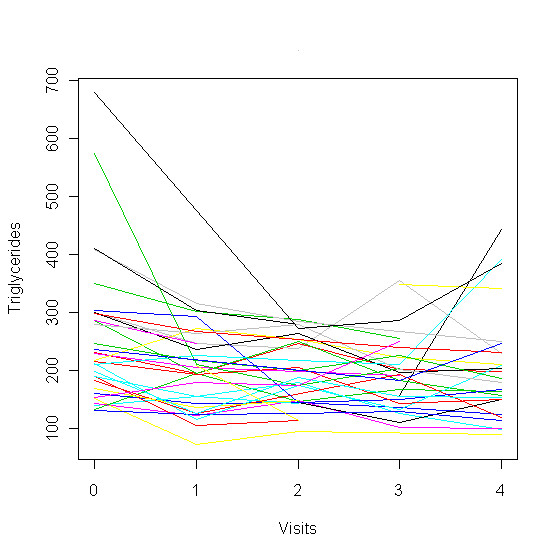
**Trajectories of Triglycerides in individual patients**. The pattern in this figure is concerned with individual changes of Triglycerides during the intervention. Each line displays the individual change of Triglycerides.

During the time course of the intervention there were no significant changes in any of the lipid parameters analyzed (Table 2). In particular, there was no increase in LDL-cholesterol. After splitting the patient group according to pretreatment, there were also no significant differences in any lipid parameters in the two groups (Table 3). The individual time course of triglycerides during the study as shown in Figure 4 exhibited a consistent decline. Three patients showed an increase of their triglycerides between the 3rd and 4th follow up visit.

**Table 3:** Results at 24 weeks split by pretreatment

	Whole	Group n = 40	Insulin	Group n = 16	OAD †	Group n = 24
	Mean	Std	Mean	Std	Mean	Std
HbA1c	6.7	0.8	6.7	0.7	6.7	0.9
Body weight, kg	101.3	17.8	94.7	17.5	105.3	17.1
Cholesterol	193.0	32.7	183.4	28.4	197.4	34.2
Triglycerides	198.8	99.6	177.0	57.0	208.7	104.5
HDL-cholesterol	54.0	17.6	56.3	10.8	53.0	20.1
LDL-cholesterol	101.8	29.3	96.2	24.4	104.6	31.7

†OAD: Oral Antidiabetic Drug

#### Treatment satisfaction

The majority of patients accepted the new treatment option as shown in Table 4. Allmost all patients indicated strong treatment satisfaction on the Likert scale with respect to blood glucose values and body weight. Restriction of carbohydrates in conjunction with liraglutide and metformin treatment, offering thereby the option to avoid insulin, was very well accepted by the majority of the patients.

**Table 4:** Treatment satisfaction

	Score						
	1†	2	3	4	5	6	7‡
Treatment satisfaction overall	0	0	0	0	5	10	25
Satisfaction with weight	0	0	0	4	6	15	18
Satisfaction with blood glucose	0	0	1	2	8	13	19
Avoidance of carbohydrates	1	1	2	9	10	15	5
Maintaining new therapy	0	0	0	4	7	11	18
Permanent reduction of carbohydrates	0	0	0	2	7	15	16

†1 = strongly disagree

‡7 = completely agree

#### Medication changes

According to the study protocol, all patients were on two oral anti-diabetic drugs or on insulin at baseline. Table 5 shows the changes in medications for those patients who were taking insulin at baseline. 13 patients out of 14 insulin-treated patients who completed the study were able to stop insulin, including those who had been on large doses of insulin; one patient had been injecting 300 insulin units per day.

**Table 5:** Changes in medications among patients taking insulin at baseline, who completed the study

Participants	Medication at baseline	Medication at 24 weeks
1	metformin 2 g, glimepirid 3 mg, insulin 28 units	metformin 2 g, liraglutide 1.2 mg
2	metformin 2 g, glimepirid 3 mg, insulin 28 units	metformin 2 g, liraglutide 1.8 mg
3	metformin 0.85 g, insulin 140 units	metformin 1.7 g, liraglutide 1.2 mg
4	metformin 1,7 g, insulin 60 units	metformin 1.7 g, liraglutide 1.2 mg
5	metformin 1 g, insulin 100 units	metformin 1 g, liraglutide 0.6 mg
6	metformin 2 g, insulin 100 units	metformin 2 g, liraglutide 1.2 mg
7	metformin 2 g, insulin 40 units	metformin 2 g, liraglutide 1.2 mg
8	metformin 2 g, insulin 300 units	metformin 2 g, liraglutide 1.2 mg
9	metformin 1.7 g, insulin 64 units	metformin 1.7 g, liraglutide 1.2 mg
10	metformin 0.85 g, insulin 132 units	metformin 1.7 g, liraglutide 1.2 mg
11	metformin2 g, insulin 70 units	metformin 2 g, liraglutide 1.8 mg
12	metformin 1 g, insulin 72 units	metformin 1 g, liraglutide 1.2 mg
13	insulin 30 units	metformin 2 g, liraglutide 1.2 mg

## Discussion

HbA1c, which reflects cumulative metabolic control during the previous three months, is considered to be the most important predictor for microvascular outcomes. This was shown for type 1 diabetes as well as for type 2 diabetes [17,18]. The impact of metabolic control of blood glucose on macrovascular outcomes is a matter of debate. The results of the recent published ACCORD Trial showed no benefit with respect to mortality for patients with type 2 diabetes with a traget of HbA1c below 6 [19]. Because patients with type 2 diabetes exhibit high risk for cardiovascular outcomes, it is important to reach metabolic control with few side effects and low risk of hypoglycemia. People on anti-diabetic drugs will often gain body weight, further increasing risk for cardiovasuclar disease. This is especially true of insulin. In our study, using a combined approach of low-carbohydrate diet in combination with metformin and the novel substance liraglutide patients were able to achieve metabolic control; seventy-one percent of patients attained a HbA1c below 7.0%. In absolute terms, there was a drop in mean HbA1c from 9.0% to 6.7%. This is far greater than that attained by other treatment options. Most anti-diabetic oral drug treatment options will reduce HbA1c by about 1% [20]. Although insulin is claimed to have the strongest effect on metabolic control, results even with large amounts are often disappointing. The patients in this study, who were already on insulin - some patients injecting more than 150 U/day - were able to stop insulin while maintaining a level of metabolic control comparable to that in the group without insulin pretreatment. This is quite remarkable because, according to base line data, the duration time of diabetes in the insulin pretreated group was more than 10 years. Additionally, they were able to lose weight: initial results at three months were maintaining until the end of the study. The negative effect on cardiovascular risk factors such as cholesterol that is frequently claimed as a risk of low-carbohydrates was not found in this study. There was no significant change in cholesterol, HDL-cholesterol and LDL-cholesterol during the course of the study.

HbA1c declined in the first 4 weeks by 13% whereas body weight declined only by 5%. At week 8, HbA1c had already reached its final reduction of 23% comparable to 26% at week 24. By comparison, body weight declined 6% at week 8 and had reached 13% at week 24. Because HbA1c is the average of glucose concentrations during the previous 3 months, the large drop within the first 4 weeks is not seen in this slowly reacting parameter and reflects an abrupt improvement and normalization of glucose concentrations. This largely weight- independent improvement in metabolic control is in line with previous work showing that weight change and glycemic control are not serially linked [12,21].

Patients were very satisfied with this treatment option. They were able to understand the detrimental effect on their metabolism of carbohydrates with high glycemic index and to make reasonable food choices in the future. All patients were willing to continue with this treatment option and did not have the feeling of being on a diet. Most of the patients had had diabetes for quite a long time and were relieved to get off insulin and have the chance to lose weight.

Limitations of our study include the absence of a control group, patients either treated conventionally with intensified insulin treatment or with carbohydrate-restricted diet or metformin or liraglutide alone. Due to limited time, logistic and funding resource, this study design was not possible. Drop-out rate was quite low, but was not zero. This may give rise to the possibility of bias in favor of treatment effects. In addition, no food frequency questionnaires were taken during the time course of the study. We are not sure that two patients who showed an upward trend of their HbA1c at 6 months really followed the principle of the diet (Figure 2). Our study was performed in an outpatient setting in the world of private praxis. For logistic reasons laboratory measurements could not be done centrally. Each office had its method for running the analysis. With respect to HbA1c the current international standard will make measurements between offices comparable. Beyond the proof-of-principle, which showed a remarkable treatment effect, this study is able to do power calculation for further randomized trials comparing this novel method with standard treatment.

The beneficial effect of low-carbohydrate diets on glucose metabolism in patients with type 2 diabetes will enable reduction in medication. This applies as well to anti-hypertensive drugs, which were also often reduced. Especially noteworthy is this treatment option for patients with short duration of diabetes. Even patients with newly diagnosed diabetes type 2 showing HbA1c values around 9% can be treated with low-carb diets alone or in conjunction with metformin. In our study most patients had a long lasting disease and had been treated for several years with standard treatment including diabetes education, low fat diets with high complex carbohydrate, but had been unable to achieve metabolic control. Switching to this alternative diet regimen was seen as providing relief.

## Conclusions

In summary, dietary carbohydrate restriction in conjunction with metformin and liraglutide treatment is an effective treatment option for patients with advanced diabetes who are scheduled to switch to insulin or who are in need of intensified insulin treatment to regain metabolic control. During this 24-week outpatient proof-of-principle study, patients showed a significant treatment effect with a majority of patients reaching HbA1c-values below 7%. The treatment effect shown will form the basis for further power calculation for a randomized intervention trial comparing this new treatment option with standard methods.

## Competing interests

The authors declare that they have no competing interests.

## Authors' contributions

JEM planned the study and was responsible for designing data management and statistical analysis. He had full access to all the data in the study and takes responsibility for the integrity of the data. DSM was responsible for designing and supplying educational material. All five participating offices provided patients and discussed the results. JEM and DSM drafted the manuscript. All authors read and approved the final manuscript.
